# Breast Cancer; Discovery of Novel Diagnostic Biomarkers, Drug Resistance, and Therapeutic Implications

**DOI:** 10.3389/fmolb.2022.783450

**Published:** 2022-02-21

**Authors:** Samia Afzal, Muhammad Hassan, Safi Ullah, Hazrat Abbas, Farah Tawakkal, Mohsin Ahmad Khan

**Affiliations:** ^1^ Centre of Excellence in Molecular Biology, University of the Punjab, Lahore, Pakistan; ^2^ Department of Genetics, Hazara University, Mansehra, Pakistan

**Keywords:** diagnostic biomarkers, circular RNAs, miRNAs, drug resistance, breast cancer

## Abstract

Breast cancer is the second most reported cancer in women with high mortality causing millions of cancer-related deaths annually. Early detection of breast cancer intensifies the struggle towards discovering, developing, and optimizing diagnostic biomarkers that can improve its prognosis and therapeutic outcomes. Breast cancer-associated biomarkers comprise macromolecules, such as nucleic acid (DNA/RNA), proteins, and intact cells. Advancements in molecular technologies have identified all types of biomarkers that are exclusively studied for diagnostic, prognostic, drug resistance, and therapeutic implications. Identifying biomarkers may solve the problem of drug resistance which is a challenging obstacle in breast cancer treatment. Dysregulation of non-coding RNAs including circular RNAs (circRNAs) and microRNAs (miRNAs) initiates and progresses breast cancer. The circulating multiple miRNA profiles promise better diagnostic and prognostic performance and sensitivity than individual miRNAs. The high stability and existence of circRNAs in body fluids make them a promising new diagnostic biomarker. Many therapeutic-based novels targeting agents have been identified, including ESR1 mutation (DNA mutations), Oligonucleotide analogs and antagonists (miRNA), poly (ADP-ribose) polymerase (PARP) in BRCA mutations, CDK4/6 (cell cycle regulating factor initiates tumor progression), Androgen receptor (a steroid hormone receptor), that have entered clinical validation procedure. In this review, we summarize the role of novel breast cancer diagnostic biomarkers, drug resistance, and therapeutic implications for breast cancer.

## 1 Introduction

Breast cancer is a kind of cancer that affects mostly females and is a primary factor of mortality worldwide (Wu and Chu, 2021). It is a heterogeneous disease with six distinct molecular subtypes: luminal A (progesterone receptors (PR)+, estrogen receptor (ER)+, Human epidermal growth factor receptor 2 (HER2)-, and Ki67), luminal B (ER+, HER+/−, and Ki67+), human epidermal growth receptor 2(HER2)+, basal-like subtype (ER-, PR- and HER2−) normal breast like and claudin low type (where low expression of cellular adhesion genes can be detected) (Perou et al., 2000). Breast cancer initial detection and monitoring are two significant treatments that enhance therapy results and provide patients with a positive prognosis (Pace and Keating, 2014). Mammography is a common approach for identifying breast cancer, although it has several drawbacks, such as a low sensitivity of 25%–59% for cancer detection in younger females with dense breasts. It has also recorded erroneous negative and positive findings, as well as a 1%–10% over detection rates (Bleyer and Welch, 2012; Oeffinger et al., 2015).

Biomarkers that aid in the diagnosis, prognosis, and prediction of breast cancer are essential for timely identification and appropriate control of the disease throughout treatment (Hayes et al., 2001; Weigel and Dowsett, 2010). Moreover, an increasing percentage of patients are demanding personalized or unique treatments, demanding the development of novel biomarkers for diagnostic and prognostic procedures as well as intact cells, are utilized as biomarkers in the diagnosis of cancer. Artificial intelligence technologies like machine learning have the potential to greatly enhance the existing anti-cancer medication development process. Constituents produced by cancer-affected cells or various tissues in reaction to tumors, and also physiological indicators that may be recognized by diagnostics or molecular technology, are all examples of cancer biomarkers (Loke and Lee, 2018; Voith von Voithenberg et al., 2019). Biomarkers may be used to assess the biological condition of a disorder, which can then be used to identify the type of the tumor, its progression, or therapy responses, assisting in the control of breast cancer (Wu and Chu, 2021). Because tumor cells are so heterogeneous, a singular biomarker is insufficiently sensitive or precise to effectively diagnose cancer growth and metastasis, hence a combination of biomarkers is preferred. Significant advances in genetic fingerprints and molecular signaling processes have found a variety of biomarkers in tissues and blood (liquid biopsies) that may be used to predict the likelihood of cancer spread, resurgence, therapy recommendations, prediction, and medication tolerance. Some of these biomarkers have been utilized in clinical trials, however, their sensitivity and selectivity are zero (Nalejska et al., 2014; Wu and Chu, 2021). As a result, novel and more effective biomarkers are required. Moreover, several therapeutic approaches for breast cancer are still in their early phases of development, therefore, it is vital to find precise biomarkers that may be used to help with immunotherapies (Wu and Chu, 2021)**.**

## 2 Types of Biomarkers

Larger molecules such as nucleic acids, genetic alterations, and protein molecules, as well as intact cells, are utilized as biomarkers in the diagnosis of cancer. They can be observed in blood in the form of circulating tumor cells, DNA, and RNA enabling liquid biopsies a useful clinical technique (Eccles et al., 2013; Berghuis et al., 2017; Voith von Voithenberg et al., 2019). Prognostic and predictive biomarkers are two categories of biomarkers linked to likely clinical results and therapy success in breast cancer subtypes (Fine and Pencina, 2015; Janes et al., 2015; Simon, 2015).

## 3 Role of Macromolecules in Breast Cancer Diagnosis

### 3.1 DNA

Alteration in DNA methylation is one of the major significant molecular changes in carcinogenesis. Adenomatous polyposis coli (APC) and retinoic acid receptors-2 (RARb2) methylated promoters were discovered in 93.4% and 95.6% of blood samples from females having breast cancer, respectively, but not in healthy people (Swellam et al., 2015). All methylation variations surpassed the conventional markers CEA and CA 15–3 in detecting early breast cancer, low-grade tumors, and Triple Negative Breast Cancer (NBC). Utilizing a human methylation DNA study BeadChip. Yang et al. revealed that hypomethylation of S100 calcium-binding protein P (S100P) and hyalurono glucosaminidase 2 (HYAL2) in the peripheral blood is linked with breast cancer (Yang et al., 2015; Yang et al., 2017). Both genes with lower methylation have been proven to be possible biomarkers circulating in blood, for the diagnosis of breast cancer, specifically in adolescent girls in the initial stages (Bleyer and Welch, 2012; Fleming and Powers, 2012).

The latest study has found that aberrant DNA methylation is significantly linked to breast cancer, and suggesting that DNA methylation testing can aid in predicting the prognosis of breast cancer patients. MAST1, PRDM14, and ZNF177 irregular DNA methylation variants were found and verified as prospective breast cancer molecular indicators by X Mao et al. X Mao et al. also showed that the DNA methylation range of ADCY4, CPXM1, DNM3, PRDM14, PRKCB, and ZNF177. In essence, these findings point to the development of novel epigenetic prognosis systems that might assist in the detection and prediction of breast cancer therapy (Uehiro et al., 2016). An exceptionally sensitive mobile cell-free DNA (cfDNA) system with epigenetic biomarkers and droplet digital methylation-specific PCR (ddMSP) has been created for the early diagnosis of breast cancer. Ras-specific guanine nucleotide-releasing factor 1 (RASGRF1), carboxypeptidase X (CPXM1), Hox-A10 (HOXA10), and Dachshund homolog 1 (DACH1) were the four methylation markers employed in the efficient screening method (Uehiro et al., 2016). This epigenetic-marker-based technique was able to reliably discriminate women with breast cancer from healthy controls, hinting that it might be utilized for breast cancer screening and therapy (Uehiro et al., 2016).

### 3.2 Proteins

Circulating protein has been considered as the second choice as a biological marker for the recognition and analysis of Breast cancer (BC). Blood proteomics and mass spectrometry have analyzed the systemic and comprehensive visualization of the blood proteomics pathologically and physiologically, resulting in the finding of numerous protein biomarkers in blood that can be used as effective diagnostic biomarkers in breast cancer detection. A panel of trefoil factor (TFF) 1, TFF2, and TFF3 have been stated as promising biomarkers for BC screening as they can express specific proteins differentially in the serum of BC patients that cannot be produced by healthy cells (Ishibashi et al., 2017). The two well-known groups such as Cks of intermediate filaments and glycoprotein (MUC) family can produce several classical breast cancer biomarkers. The CA 15–3 assay, for example, is currently used for tracking purposes in treatment (Duffy, 2006), while CKs have been proposed as early-stage BC markers, but their efficacy is masked due to poor sensitivity (Levenson, 2007). The serum epithelial membrane antigen/CK1 concentration ratio is recommended as possible diagnostic marker, especially for initial stage breast cancer diagnosis. The diagnostic capability of this new combination was assessed better than CA 15–3 (Attallah et al., 2014).

Besides, some other proteinaceous biological markers with promising diagnostic capability have been revealed by ELISA. Among these proteins, a single diagnostic marker model of pleiotrophin (PTN) (Ma et al., 2017a), and double diagnostic marker models such as integration of microRNA (miRNA) miR-127-3p with human epididymis secretory protein 4 (HE4) (Lu et al., 2017), human anterior gradient (AGR) 2 with AGR3 (Garczyk et al., 2015) and vascular endothelial growth factor (VEGF) with CA 15–3 (Ławicki et al., 2016). Serum apolipoprotein C-I (apoC-I) has demonstrated promise results in prognosis and diagnosis of triple-negative Breast cancer (TNBC), as it could distinguish TNBC from non-TNBC cases by greater ApoC-I mRNA and protein expression in the former when compared to both non-TNBC affected individuals and controls (Song et al., 2016).

### 3.3 Autoantibodies

Autoantibodies are reported as another approach used as diagnostic biomarkers with the potential of multiple targets, short time-frames, and minimalist hardware (Soler et al., 2016). The associated antigens of these antibodies are synthesized by the body’s cells; in healthy cells, it is expressed at a modest level, while it is overexpressed in malignant cells. The immune system determines this expression, using toll-like receptors (TLRs) for the innate response, thus reverting tumor growth. MUC1, an integral membrane protein, is overexpressed in 90% of adenocarcinomas and has been linked to tumor aggressiveness (Zaenker et al., 2016). Antibodies that target oncogenic and tumor suppressor proteins are considered as the significant diagnostic biomarkers for the efficient detection of breast cancer. The presence of autoantibodies before the medical diagnosis of the disorder in paraneoplastic syndrome and systemic autoimmune diseases has raised the prospect that the medical diagnosis of BC could also be carried out through detection of auto-immunoglobulins (Fernández Madrid, 2005). As a result, extensive research has revealed the detection of tumor-associated antigens by autoantibodies that were detected in the patient blood sample. The integration of modern proteomics, advanced genomics, high-throughput technology, and traditional immunological approaches has significantly aided advancement in this area (Fernández Madrid, 2005).

### 3.4 miRNA

miRNAs are single-stranded, non-coding, small (20–25 nucleotide) RNAs that suppress the post-transcriptional expression of specifically selected genes via mRNA breakdown or mRNA expression. The detection and persistence of miRNAs in the bloodstream and their role in diagnostic and therapeutic approaches have been studied thoroughly (Schwarzenbach et al., 2011). Since miRNAs are soluble and observable in cancer cells (Shen et al., 2013), blood, plasma, and patients’ saliva, they can be used as biological markers for non-invasive early diagnosis, detection, and treatment of breast cancer (Mitchell et al., 2008; Schwarzenbach et al., 2014). miRNAs are released into the bloodstream by apoptotic cells (Schwarzenbach et al., 2011). miRNAs can travel through the bloodstream in two forms: cell-free (Ago2-related) or embedded in membrane vesicles, microvesicles, or exosomes (Simpson et al., 2009). miRNAs profiling studies can classify dysregulated miRNAs and categorize patients of breast cancer for therapies, highlighting their potential to be used as a predictive and therapeutic biomarker (McGuire et al., 2015). Furthermore, like oncogenes (oncomiRNAs) and tumor suppressors, unregulated miRNA is responsible for tumor growth, development, cell death, invasion, and cell proliferation. Recently, numerous suspected candidates, for example, miR-221, miR-21, and miR-145, have appeared in the blood serum or plasma of BC-affected individuals. Blood-based identities containing miR-221 and/or miR-21 have been shown to have higher diagnostic susceptibility than CEA and CA 15–3 for all stages of cancer, distinguishing BC subjects from patients with benign tumors and healthy people and doing better in distinguishing the TNBC subtype from healthy subjects (Gao et al., 2013; Motawi et al., 2016; Thakur et al., 2016). Exosomal miR-21 expression has also been shown to be elevated in Breast cancer patients’ plasma-derived exosomes. The exosomal miR-21 and miR-1246 form modest diagnostic (Hannafon et al., 2016).

In terms of diagnosis, Iorio et al. (2005) discovered a 13-miRNA hallmark that could differentiate affected Breast cancer from healthy breast tissues with 100% precision. Blenkiron et al. (2007) discovered 133 miRNAs with abnormal expression patterns in breast tumor tissues irrespective of normal breast tissues. Despite the discovery of miRNAs with abnormal expression in BC tissues, there are still inconsistencies between the variously identified miRNA signatures. Roth et al. introduced single circulating miRNAs as diagnostic and prognostic instruments after discovering miR-155 in the serum of Breast cancer affected individuals but not in stable controls, and Heneghan et al. identified elevated miR-195 activity in the bloodstream of only affected subjects with BC (Heneghan et al., 2010; Roth et al., 2010). Some miRNAs, such as miR-21and miR-29a or 4-miRNA signature, have been found in the plasma of BC patients (miR-222, miR-16, miR-25, and miR-324-3p) (Wu et al., 2011; Hu et al., 2012).

A vast number of other miRNAs have been reported that overexpress in Breast cancer a few examples are miR-29a, miR-146a, miR-373, miR589, miR-221/222 cluster, miR-9, miR10b, miR-96, miR-181, miR-375, and miR-520c. These miRNAs are linked with treatment, diagnosis, and prognosis of Breast cancer (Polytarchou et al., 2012; Khoshnaw et al., 2013; Sandhu et al., 2014).

### 3.5 CircRNAs as Diagnostic/Prognostic Markers

Circular RNAs (circRNAs) are recently identified non-coding RNAs that are classified as tiny endogenous RNAs with a broad distribution, various forms, and several regulatory applications (Ashwal-Fluss et al., 2014). Sanger et al. (1976) were the first to find cricRNA in the viroids. Up till now, a large number of circRNAs have been found in various cell lines and organisms (Bleyer and Welch, 2012; Pace and Keating, 2014; Oeffinger et al., 2015), namely protozoa, fungi, worms, plants, fish, mice, insects, and humans (Wang et al., 2014; Westholm et al., 2014). CircRNAs are present in large numbers, about 1/8th of the human genome’s transcriptome can generate observable circRNAs, and their expression levels are much more than tenfold higher than that of the comparable linear mRNAs (Salzman et al., 2012; Jeck et al., 2013). Furthermore, as a result of their covalent closed-loop configuration and absence of free terminal ends, circRNAs are much more stable unlike linear RNAs, conferring susceptibility to deterioration by endonuclease R (RNase R) (Chen and Yang, 2015). CircRNAs can also be used to distinguish and recognize various tumor types due to their ability to express particular cell types, tissues, and developmental stages, as well as the fact that different subtypes of circRNAs can be generated (Salzman et al., 2013; Smid et al., 2019). Given the above, we conclude that circRNAs have growing research potential. Various biochemical roles of circRNAs have been discovered as science has progressed. CircRNAs may serve as “sponges” for microRNAs (miRNAs), influencing the role of miRNA target genes (Hansen et al., 2013). Furthermore, circRNAs can be attached to various RNA binding proteins (RBPs), influencing the role of the parental genes (Zeng et al., 2017; Kristensen et al., 2019). Surprisingly, provided evidence suggests that circRNAs may express proteins/peptides playing a role in tumor development and progression (Legnini et al., 2017; Zheng et al., 2019). CircRNAs’ special properties and biological roles show the role of circRNAs in tumor growth, replication, metastasis, invasion, and drug tolerance, implying that circRNAs can be used as biological markers and tumor therapeutic goals (Liu et al., 2018a; Kun-Peng et al., 2018). Circular RNAs can avoid exonuclease-induced degradation and are more soluble in blood or plasma than linear RNAs because of their closed continuous loop structure (Alhasan et al., 2016). It has been shown that ncRNAs, such as miRNAs and non-coding RNAs, can serve as reliable biological markers for hepatocellular carcinoma (Li et al., 2015). CircRNAs are regaining attention among researchers as high-throughput sequencing and bioinformatics technology improves. Due to their stability and tissue specificity, circRNAs have been established as suitable biological markers for the diagnosis of gastric cancer (Simon, 2015), hepatocellular carcinoma, and other cancers (Qin et al., 2016). Evidence have revealed that circRNAs can cause tumorigenesis, providing a new approach for identifying diagnostic biomarkers (Kulcheski et al., 2016; Zhu et al., 2017).

Using the circRNA microarray method, Lu et al. determined the circRNA expression pattern in breast cancer and normal tissues and discovered that hsa circ 103110, hsa circ 104689, and hsa circ 104821 levels were upregulated in breast cancer cells with area under the curve (AUC) value of 0.63 (0.52–0.74), 0.61 (0.50–0.73), and 0.60 (0.49–0.71) respectively, while hsa circ 006054, hsa circ 100219, and hsa circ 406697 were downregulated with the area under the curve (AUC) value of 0.71 (0.61–0.81), 0.78 (0.69–0.88) and 0.64 (0.52–0.75) respectively. As a result, mixing hsa circ 006054, hsa circ 100219, and hsa circ 406697 yielded successful diagnostic results as mentioned in table 1 (Lü et al., 2017). Similarly, Yin et al. found that in the plasma of breast cancer patients, 19 circRNAs were upregulated and 22 were downregulated as opposed to stable controls (Maselli et al., 2019). Further investigation found that the plasma hsa circ 0001785 had a higher diagnostic accuracy than CEA and CA15-3. Furthermore, hsa circ 0001785 plasma levels were correlated with histological grade (*p* = 0.013), TNM stage (*p* = 0.008), and remote metastasis (*p* = 0.016), indicating a possible biomarker for breast cancer diagnosis. The expression levels of hsa circ 0001785 were shown to be lower in post-operative BC patients’ plasma samples relative to pre-operative patients (Sarkar and Diermeier, 2021).

**TABLE 1:** Regulation pattern of different circRNAs in cancer lesions.

Regulation pattern	Types of CircRNAs	Value of area under the curve (AUC)
Upregulation in cancer lesions	hsa circ 103110	0.63 (0.52–0.74)
	hsa circ 104689	0.61 (0.50–0.73)
	hsa circ 104821	0.60 (0.49–0.71)
Downregulation in cancer lesions	hsa circ 006054	0.71 (0.61–0.81)
	hsa circ 100219	0.78 (0.69–0.88)
	hsa circ 406697	0.64 (0.52–0.75)

#### 3.5.1 Limitations of Circulating miRNA as a Diagnostic Biomarker

The development of a precise and effective biomarker for breast cancer diagnostic approaches is challenging at every step ranging from sample collection to data processing (Witwer, 2015). For example, the low abundance of circulating miRNAs as diagnostic biomarker hinders their detection using microarray-based miRNA profiling techniques. Modified strategies can be adopted to reduce the limitations; such as miRNA isolation before expression profiling (Hamam et al., 2016). Another issue is sample collection which can be resolved by serum selection to avoid limitations of excluding a large number of samples. Because recent studies reported a high level of circulating miRNA in serum than in plasma (Wang, 2012). A recent study identified the fluctuations in circulating miRNA levels in response to chemotherapy. This drawback can be eliminated by collecting blood samples before chemotherapy (Diener et al., 2015).

### 3.6 Exosome

Exosomes are extracellular membrane-bound vesicles that are nano-sized (30–100 nm) and actively released by cancer cells and neighboring cells present in the tumor microenvironment (TME) (Taylor and Gercel-Taylor, 2013; Gajos-Michniewicz et al., 2014). They are surrounded by a lipid bilayer composed up of phosphoglycerides, ceramides, sphingolipids, and cholesterols (Vlassov et al., 2012) and comprise a diverse array of molecules such as DNA, sugars, proteins, peptides, lipids, mRNAs, miRNAs, as well as other types of ncRNAs (Moreno-Gonzalo et al., 2014). Exosomes like miRNAs, are present in a variety of human bodily fluids, including blood, sweat, urine, and breastfeeding (Vlassov et al., 2012; Raposo and Stoorvogel, 2013).

Exosomes can facilitate tumor development, angiogenesis, immunosuppression, and metastasis by promoting intermodulation between tumor cells and healthy or cancer-affected stromal cells (Alderton, 2012; Moon et al., 2016a). The surface proteins present on circulating extracellular vesicle (EVs), developmental endothelial locus-1 protein (Del-1) (Moon et al., 2016a), and fibronectin (Moon et al., 2016b), are promising materials for cancer detection. Fibronectin, a matrix protein present extracellularly, binds to several integrins and activates a variety of signaling proteins, including FAK, Src, and Akt (Moon et al., 2016b). Fibronectin levels rose dramatically (*p* < 0.0001) throughout all phases of breast cancer and went back to normal after the tumors were removed. The clinical diagnostic efficacy for fibronectin recognition outside the cellular vesicles was better than that in plasma (Moon et al., 2016b). This underscores the significance of extracellular matrix proteins in breast cancer. The high amount of Del-1 in patients’ circulating exosomes (*p* 0.0001) resulted in excellent diagnostic success in distinguishing patients with early-stage breast cancer from the control system (Moon et al., 2016a).

## 4 Role of Biomarkers in Drug Resistance

Chemotherapy treatment has a significant role in the prevention of breast cancer recurrence and spreading (Liu et al., 2018b). But the main problem of this method is Chemo-therapeutic resistance, hsa-circ 0006528 is upregulated in breast cancer cells resistant to Adriamycin resistant breast cancer (ADM), presumably by the circ. pathway. Axis of RNA/miR-7-5p/Raf1 (Guo et al., 2020). Low concentrations of miR-7 expression have long been associated with resistance to breast cancer chemotherapy. Another research of ADM-resistant breast cancer discovered that circKDM4C downregulation inhibited tumor proliferation and alleviated ADM resistance by controlling the miR-548p/PBLD axis (Ma et al., 2019; Yang et al., 2020).

Furthermore, the level of expression of circMTO1 (hsa circ 007874) in monastrol-resistant breast cancer cell lines is substantially lower than in monastrol-sensitive breast cancer cell lines, and uncontrolled expression of circMTO1 will reverse monastrol resistance through the circRNA/TNF receptor-associated factor 4 (TRAF4)/Eg5 pathway. Furthermore, Ma et al. discovered that circMOTL1, which could play an important role in breast cancer cell PTX resistance by controlling the AKT pathway, encouraging anti-apoptotic protein expression, and impeding pro-apoptotic protein synthesis, is found to be elevated in breast cancer (Greene et al., 2019). Yang et al. reported that in Breast cancer cells, the expression of circ-ABCB10 is increased. Through the let-7a-5p/DUSP7 axis, Circ-ABCB10 regulates PTX resistance, apoptosis, invasion, and autophagy in breast cancer cells (Wu et al., 2019).

The impact of Erα36 on the oncogenesis of breast and drug resistance was assessed by Pangano et al. (Yin et al., 2018). Tamoxifen, reported as anti-estrogen, has been shown to act as an agonist of ER36 to proliferate, invade, and metastasize breast cancer cells (Yin et al., 2018), which explains why many breast cancer patients develop drug resistance to anti-estrogens that block the signaling pathways mediated by ER36. A serum autoantibody was recently discovered that functions against ERα in a wide proportion of patients affected by breast cancer and was shown to cause ER36, leading to tamoxifen tolerance (Maselli et al., 2016; Maselli et al., 2019).

### 4.1 Role of miRNA

There are several miRNAs whose expressions were downregulated in drug resistance BC. In BC-affected tissues and cells resistant to taxol, the increased expression of nuclear receptor co-activator 3 (NCOA3) results in decreased expression of miR-17 and miR-20b. This shows that these three NCOA3, miR-17, and miR-20b may function as active biological markers and therapeutic targets in breast cancer resistance to taxol (Ao et al., 2016).

**FIGURE 1:**
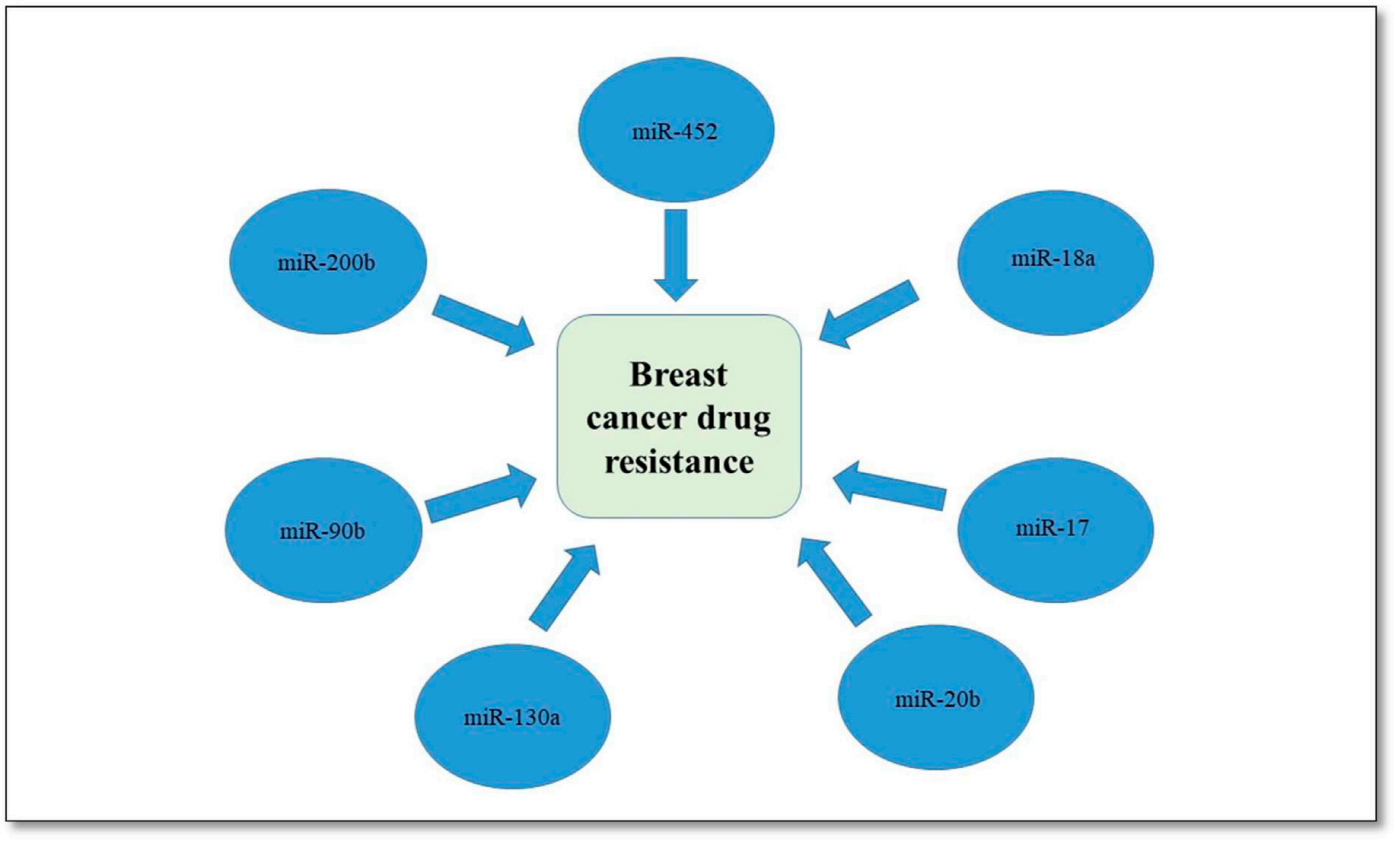
Role of different miRNA in drug resistance.

Another study reported the overexpression of miR-18a in TNBC cells patients who had received neoadjuvant paclitaxel, thus inhibiting dicer expression and increasing paclitaxel resistance in TNBC cells (Sha et al., 2016). There are four miRNAs; miR-90b, 130a, 200b, and 452 that can regulate drug-related cellular pathways, thus leading to chemoresistance as shown in Figure 1 (Jayaraj et al., 2019).

### 4.2 Role of circRNAs

In adriamycin-resistant cell lines and tissues sensitive to Adriamycin, a high-level expression of hsa_circ_00006528 was discovered by Gao et al. (Ma et al., 2017b) by using circRNA microarray expression profiles. The hsa_circ_00006528-miR-7–5p-Raf1 axis has a regulatory effect on breast cancer resistance to adriamycin. This highlights the possibility of the use of hsa_circ_00006528 in controlling the factor of drug resistance. This concludes that for breast cancer treatment, circRNAs come up with novel and reliable therapeutic strategies (Ma et al., 2017b).

has-circ_00006428, a circular RNA, can be used as a promising therapeutic candidate due to its role in reducing drug resistance. This statement can be supported by its overexpression in Adriamycin-resistant cells while expression in Adriamycin-sensitive cells. Besides, the regulatory role of hsa_circ_00006528-miR-7-5p-Rafl axis in Adriamycin resistant breast cancer was revealed (Ma et al., 2017b).

## 5 Therapeutic Implications

### 5.1 CircRNAs as Therapeutic Targets in Breast Cancer

New progress in RNA-based therapies plus aberrant expression of circRNAs in breast cancer makes them more potential therapeutic sites (Lei et al., 2019). One solution may be to create synthetic circRNAs with several binding sites for certain oncogenic proteins or miRNAs that could be inserted exogenously to restore the natural regulatory network in the cell and reduce cancer development (Tay et al., 2015; Liu et al., 2018c). On the other hand, endogenous circRNAs can be useful for the treatment of cancer (Dragomir and Calin, 2018). Any of the tumor-suppressor circRNAs discussed above are prime candidates for further development as therapeutic instruments. CircFOXO3, circCCNB1, circKDM4C, circFBXW, and circTADA2A were all shown to be downregulated in patient samples, related to a bad diagnosis and treatment, and biologically linked to cancer etiology (Meganck et al., 2018). CircRNA overexpression constructs transferred by adeno-associated virus (AAV) vectors, which do not incorporate into the genome and are presently used in clinical studies, are recently used as a therapeutic solution. On another side, tumorigenic circRNAs, such as the TNBC-specific circAGFG1 and circANKS1B, may be used as new therapeutic targets for TNBC, which currently have few treatment choices and a weak prognosis (Bianchini et al., 2016). To target overexpressed circRNA expression therapeutically, effective approaches have been designed, including degradation mediated by siRNA, shRNA, or altered antisense oligonucleotides (ASOs) complementary to the back-splice junction (Cortés-López and Miura, 2016; Santer et al., 2019). More steady knockout methods, such as CRISPR/Cas genome editing, have also been evaluated. The CRISPR/Cas13 system is a new addition that achieves circRNA silencing by attacking Cas13 to the circRNA’s back-splice junction via a specific guide RNA that can differentiatedifferentiate between linear transcripts and circRNAs (Li et al., 2021). Cas13d, a small version of Cas13 (Konermann et al., 2018), in particular, may be packaged into an AAV vector for transmission into primary cells and rodents (Konermann et al., 2018; Zhang, 2021). However, since the side effects of Cas13 expression are currently uncertain, the drawbacks of this system in a clinical sense are not known (Li et al., 2021).

## 6 Homologous Recombination Deficiency in Breast Cancer Genes

Genomic mutations and instability are the attributions of human cancers that occur due to defective DNA repair mechanisms. One such DNA repair process is homologous recombination (HR), which facilitates the repair of double-strand breaks and interstrand cross-links (Li and Heyer, 2008). Mutations in BRCA1 and BRCA2 genes are centrally involved in homologous recombination (HR), DNA damage repair, and cell cycle checkpoint regulation (Joosse, 2012). About 5%–10% of breast cancers are associated with inherited mutations in BRCA1 and BRCA2 genes (Institute, 2013). A recent study reported the significance of germline mutations of BRCA1 and BRCA2 by determining their sensitivity to platinum-based chemotherapy and PARP inhibitors (Von Minckwitz, 2014). HRD-mutational signatures are clinically associated with platinum-based chemotherapy in the advanced–stage of breast cancer (Davies et al., 2017).

## 7 Conclusion

Breast cancer is the second mostly reported cancer in women showing a high mortality rate worldwide annually. Early diagnosis and prognosis can control its fatality rate up to some extent. No single biomarker is involved in its diagnosis but a group of multiple diagnostic biomarkers plays a key role in its detection, prognosis, and treatments. Several macromolecules are reported as the significant diagnostic biomarkers including circular RNA, miRNA, DNA, protein, exosomes, and antibodies. Identification of these macromolecules can help in the detection of cancer. DNA methylation and miRNA profiling are the prominent approaches through which breast cancer can be identified.
